# Enhanced Solubility of Ibuprofen by Complexation with β-Cyclodextrin and Citric Acid

**DOI:** 10.3390/molecules29071650

**Published:** 2024-04-06

**Authors:** Tsveta Sarafska, Stanislava Ivanova, Todor Dudev, Christo Tzachev, Vesselin Petrov, Tony Spassov

**Affiliations:** Faculty of Chemistry and Pharmacy, Sofia University “St. Kl. Ohridski”, 1164 Sofia, Bulgaria; nhtth@chem.uni-sofia.bg (T.S.); nhtsi@chem.uni-sofia.bg (S.I.); ohtttd@chem.uni-sofia.bg (T.D.); ohtct@chem.uni-sofia.bg (C.T.); fhvp@chem.uni-sofia.bg (V.P.)

**Keywords:** Ibuprofen, cyclodextrin, citric acid, inclusion complex, solubility, dissolution rate

## Abstract

The ability of β-CD to form inclusion complexes with ibuprofen (IBU) and at the same time to make a two-phase system with citric acid was explored in the present study for achieving improved solubility and dissolution rate of IBU. Mechanical milling as well as mechanical milling combined with thermal annealing of the powder mixtures were applied as synthetic methods. Solubility and dissolution kinetics of the complexes were studied in compliance with European Pharmacopoeia (ICH Q4B). β-CD and citric acid (CA) molecules were shown to interact by both ball milling (BM), thermal annealing, as well as BM with subsequent annealing. Complexes were also formed by milling the three compounds (β-CD, CA and IBU) simultaneously, as well as by a consecutive first including IBU into β-CD and then binding the formed β-CD/IBU inclusion complex with CA. As a result, ternary β-CD/IBU/CA complex formed by initial incorporation of ibuprofen into β-CD, followed by successive formation of a two-phase mixture with CA, exhibited notably improved dissolution kinetics compared to the pure ibuprofen and slightly better compared to the binary β-CD/IBU system. Although the addition of CA to β-CD/IBU does not significantly increase the solubility rate of IBU, it must be considered that the amount of β-CD is significantly less in the ternary complex compared to the binary β-CD/IBU.

## 1. Introduction

As it was shown in our recent study [1], beta-cyclodextrin (β-CD) and citric acid (CA) can interact in several ways, depending on the binding conditions. β-CD is a cyclic oligosaccharide composed of seven glucose units and has a hydrophobic cavity in its center, which can accommodate a variety of guest molecules. Citric acid (CA) is a weak organic acid commonly found in citrus fruits and often used as an acidulant in food and beverage products [2,3,4,5]. The exact geometric configuration of the β-CD/citric acid complex depends on various factors such as the concentration of the two molecules, temperature, pH, and the solvent used. In general, the complexation between beta-cyclodextrin and citric acid results in the formation of a 1:1 complex, where one molecule of β-CD encapsulates one molecule of citric acid [6,7,8,9,10]. One possible configuration is the surface complexation mode, where the citric acid molecule interacts with the outer surface of the β-CD host. In this mode, the citric acid molecule can form hydrogen bonds and other non-covalent interactions with the hydroxyl groups on the outer surface of the beta-cyclodextrin molecule [11].

Ibuprofen, which is a carboxylic acid, is poorly soluble in water. The absorption of such drugs into the blood is typically a dissolution rate-limited process. The formation of host–guest complexes is a major method of improving the solubility of poorly water-soluble substances. β-CD, with its crossed cone structure characterized by a hydrophobic cavity and hydrophilic exterior surface, is well suited and used as a “host” [12,13,14].

Ibuprofen belongs to class II of the Biopharmaceutical Classification System (BCS) and is characterized by high permeability through mucous membranes and pH-dependent solubility [15]. As a free acid in conventional solid dosage forms, IBU has slow and sometimes incomplete absorption [16], showing limited solubility in the stomach at low pH [17]. The rate of absorption is limited mainly by the rate of dissolution [18]. In order to improve the dissolution rate of IBU in the stomach, salt conjugates are most widely used, e.g., IBU-lysine or IBU-arginine which are included as active substances in many OTC (over the counter) products on the European and American markets.

The undoubted benefits of using lysinate or arginate salt conjugates are offset by the large stoichiometric amounts required of the conjugate, for example 400 mg of IBU requires an additional 350–400 mg of arginine or lysine. The latter makes tablets and capsules more expensive (2–3 times), more difficult to formulate, and for patients to swallow. On the other hand, the amino acids arginine and lysine have disadvantages such as drug interactions with anticoagulants and anti-platelet drugs, herbs and supplements, blood pressure drugs, diabetes drugs, nitrates, potassium-sparing diuretics and pregnancy category C, and renal dysfunction, including Fanconi syndrome and renal failure [19,20].

Hence, searching for other technological approaches related to drug formulations based on the IBU free acid form is reasonable and promising.

Another interesting approach to increase the solubility of small, water-soluble substances is the formation of eutectic mixtures. These mixtures are characterized by a lower melting temperature, which results in higher solubility in aqueous media [21,22]. Increasing the solubility rate of poorly water-soluble drugs can be achieved by crosslinking the formed host–guest complex. Polycarboxylic acids and in particular citric acid are popular crosslinking agents used for esterification and crosslinking of polysaccharides such as starch, cellulose, as well as polyhydroxy compounds such as glycerol, sorbitol, and cyclodextrins. Better solubility in water of ibuprofen was reached also by forming ionic pairs of ibuprofen with the biocompatible counterions of L-valine alkyl ester, where the alkyl chain can enhance ibuprofen transport through biological membranes [23]. An improvement in the ibuprofen dissolution rate was observed via adsorption of IBU onto spherical porous calcium silicate [24]. Enhancement of ibuprofen solubility was also observed in surfactant solutions [25].

Hydroxypropyl cyclodextrins are modified versions with enhanced water solubility and are primarily used to improve the solubility and bioavailability of poorly water-soluble drugs. Sulfobutylether cyclodextrins, with their high-water solubility and safety profile, are particularly valued in pharmaceutical formulations for reducing drug nephrotoxicity. Methylated cyclodextrins, characterized by increased lipophilicity, are utilized to solubilize lipophilic drugs, enhancing their stability and solubility. Each type of cyclodextrin offers distinct advantages, chosen based on the application’s specific requirements regarding solubility, safety, and drug interaction capabilities [26,27,28,29,30,31].

The aim of the present work is to investigate the possibility of improving ibuprofen solubility and dissolution rate by forming a two-phase system in which one of the components is β-cyclodextrin including ibuprofen, and the other-citric acid. This goal is also related to reliably elucidating the intimate mechanism of binding realized between the three compounds.

## 2. Results and Discussion

### 2.1. Theoretical Modelling

**Figure 1:**
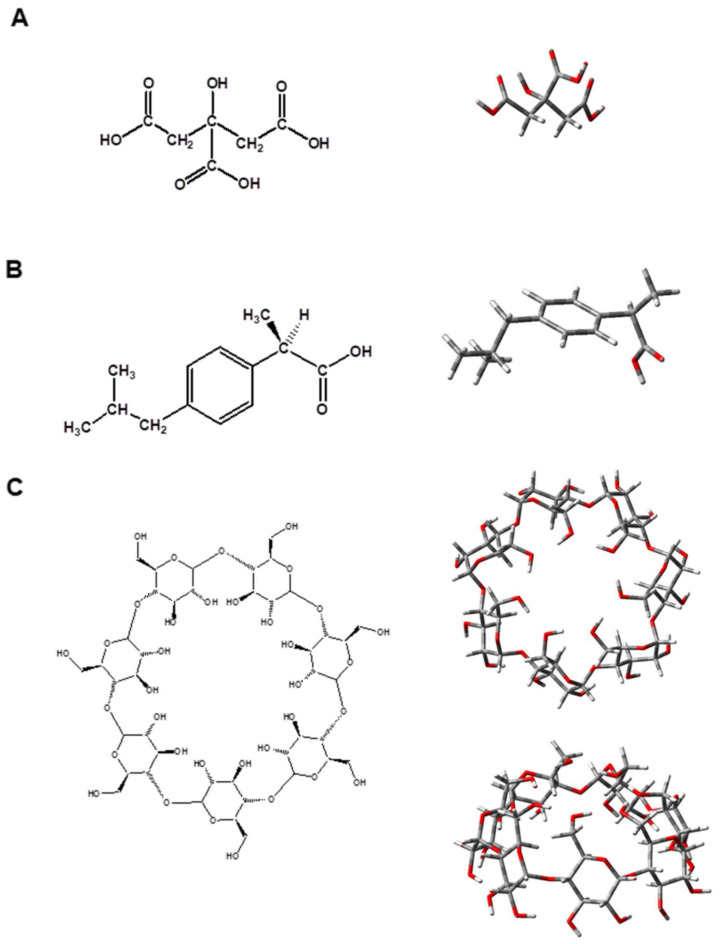
Chemical representations and M062X/6-31G* fully optimized structures of (**A**) citric acid, (**B**) R-ibuprofen, and (**C**) β-cyclodextrin (top and side view for the optimized structures). Color scheme: C–gray, H–white, O–red (structures in (**A**,**C**) were reproduced from [1]; copyright Elsevier, 2024).

The three interacting entities—citric acid (Figure 1A), ibuprofen (Figure 1B), and β-CD (Figure 1C)—were modelled explicitly. The former was considered to be in a fully protonated form. The R-enantiomer of ibuprofen was modelled and employed in the calculations as it has been found that it forms more stable inclusion complexes with the host β-CD than its S- counterpart [32]. The most energetically favorable form of the β-CD molecule was considered, i.e., the “closed” configuration characterized with internal hydrogen bonds arranged in a “tail-head” fashion of the constituent -OH groups from both the upper and lower rim of the molecule [1,33].

The geometries of the three nominal compounds as well as those of the respective inclusion complexes were optimized at the M062X/6-31G* level of theory using the Gaussian 09 package of programs [34]. Minnesota M062X functional in combination with split-valence double-ζ basis set was employed in the calculations as it has been proven to be reliable in reproducing geometrical parameters of a number of macrocyclic compounds and their inclusion complexes [32,33,35]. Vibrational frequencies (all of them positive), thermal energies, E_th_, and entropies, S, at 25 °C were evaluated at the same level of theory. The electronic energies, E_el_, were corrected at higher theoretical level (M062X/6-31+G**) via single-point calculations on the M062X/6-31G* optimized structures.

#### 2.1.1. Binding of Citric Acid and Ibuprofen to β-CD in Forming 1:1 Binary Complexes

**Figure 2:**
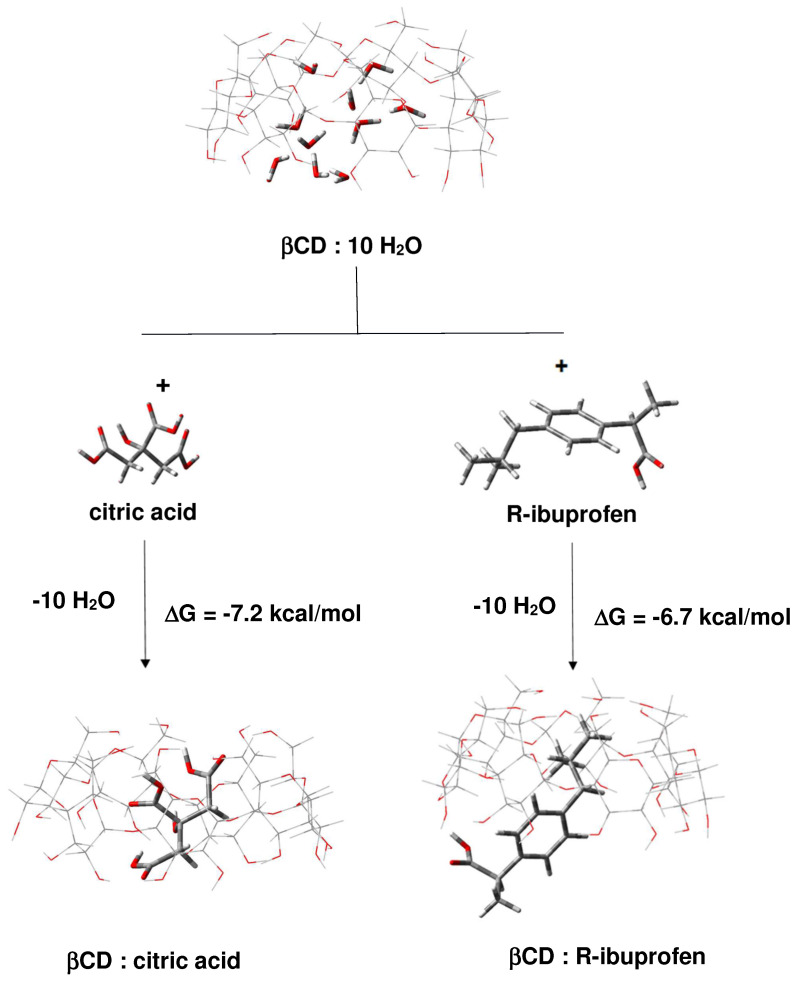
M062X/6-31G* optimized structures of reactants and resulting complexes, and the free energies of complex formation (in kcal/mol).

The thermodynamics of the interaction between the two guest molecules and β-CD, yielding 1:1 inclusion complexes, were studied. Graphically, the process is represented in Figure 2. As the calculations suggest, both citric acid and ibuprofen can interact favorably (negative Gibbs free energies) with the macrocyclic molecule anchoring at the internal cavity of the host and displacing all the interior water molecules which, as demonstrated before, had hydrated the internal cavity prior to the CA/ibuprofen complexation [33]. Note that CA exhibits (slightly) higher affinity toward the host β-CD than its ibuprofen counterpart evidenced by lower interaction ΔG for the former than the latter (left and right-hand side of Figure 2, respectively). This is most probably due to the larger number of interacting units (3-COOH and 1-OH groups) in CA compared to ibuprofen (1-COOH group).

#### 2.1.2. Formation of β-CD/CA/IBU Ternary Complexes

**Figure 3:**
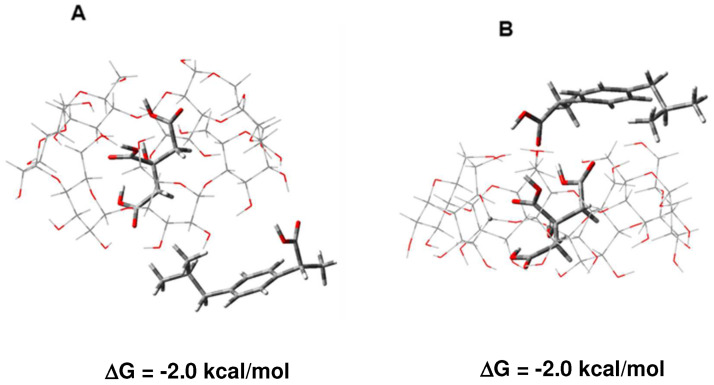
M062X/6-31G* optimized structures of β-CD/CA/IBU ternary complexes and the free energies (in kcal/mol) of complex formation (β-CD/CA + IBU → β-CD/CA/IBU). Binding of ibu-profen to lower (**A**) and upper rim (**B**).

What is the most favorable structure of the complex between β-CD, CA, and ibuprofen if the two guest molecules are present at the same time in the reaction mixture? According to the results described above, it is plausible to believe that CA, being more competitive than ibuprofen, will bind first to the host β-CD (Figure 2, left-hand side; ΔG = −7.2 kcal/mol). Then, ibuprofen can bind to the exterior of already formed β-CD/CA binary complex yielding β-CD/CA/ibuprofen ternary complex. As the calculations suggest, the process of formation of the 1:1:1 ternary complex (β-CD/CA + IBU → β-CD/CA/IBU) is thermodynamically favorable characterized with negative Gibbs free energies (Figure 3). Binding of ibuprofen to either lower (Figure 3A) or upper rim (Figure 3B) of β-CD appears equally favorable.

#### 2.1.3. Formation of β-CD/IBU/CA Ternary Complexes

**Figure 4:**
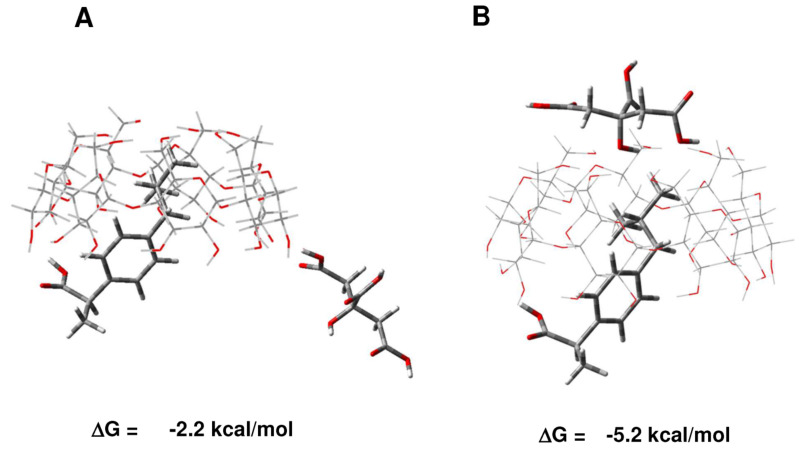
M062X/6-31G* optimized structures of β-CD/ibuprofen/CA ternary complexes and the free energies (in kcal/mol) of complex formation (β-CD/IBU + CA → β-CD/IBU/CA). ΔG between −2.2 (**A**) and −5.2 kcal/mol (**B**).

What type of complexes can be formed if the reaction takes place in a stepwise manner if ibuprofen is introduced first in the reaction medium, followed by the addition of citric acid? In such an arrangement, a binary β-CD/IBU construct will be formed first (Figure 2, right-hand side; ΔG = −6.7 kcal/mol), followed by coordination of CA to the exterior of the complex (Figure 4; ΔG between −2.2 and −5.2 kcal/mol). Both processes are thermodynamically favorable. Note that the binding of CA to the upper rim of β-CD yields a more stable complex (ΔG = −5.2 kcal/mol) than the alternative coordination to the lower rim of the host macromolecule (ΔG = −2.2 kcal/mol).

Can the incoming CA, instead of coordinating to the exterior of the β-CD/IBU construct, displace the ibuprofen from the complex and substitute for it inside the host cavity? Although CA is a bit more competitive than ibuprofen, the calculations suggest that, in the circumstances, this is not very likely, as if the CA replaces Ibuprofen, there will be a free energy gain of -0.5 kcal/mol (−7.2 + 6.7 kcal/mol). The formation of the ternary complexes β-CD/IBU/CA, however, will secure higher free energy gain of −11.9 kcal/mol (−6.7 (Figure 2 right-hand side) + (−5.2) (Figure 4B)) as compared to that of the β-CD/CA/IBU counterpart of −9.2 kcal/mol (−7.2 (Figure 2 left-hand side) + (−2.0) (Figure 3)) which appears to be more thermodynamically advantageous.

### 2.2. Experimental Evidence for the Formation of Ternary Complexes

#### Thermal, Spectral, and Microstructural Characteristics of β-CD/IBU and β-CD/IBU/CA Complexes

**Figure 5:**
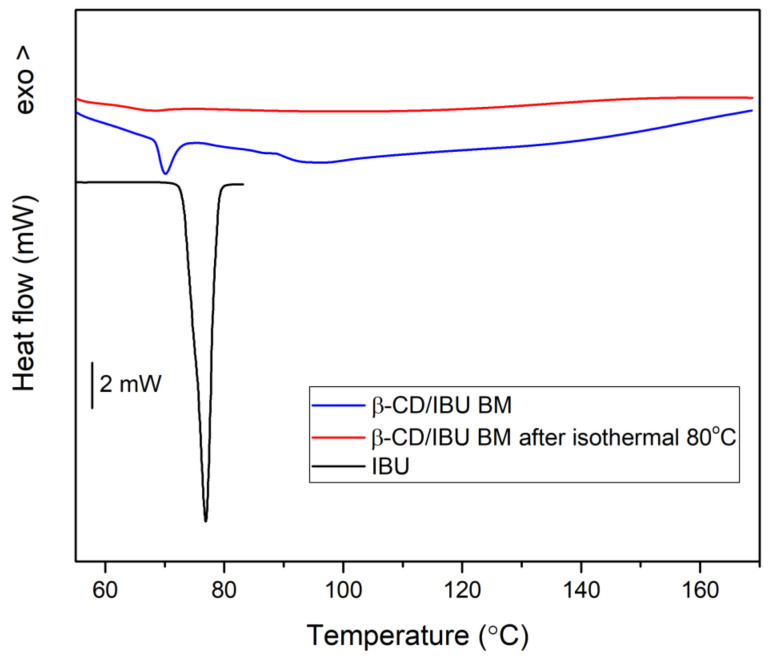
DSC scans of β-CD/IBU after ball milling and subsequent isothermal annealing.

The synthetic procedure followed the strategy applied in the theoretical study, namely, to include ibuprofen in the β-CD and then add CA to the β-CD/IBU complex. The complex between β-CD and ibuprofen was obtained through ball milling. For this purpose, β-CD and ibuprofen were mixed in a molar ratio of 1:1. The milling conditions were powder to balls 1:4 (4 balls of 1 g each to 1 g powder), milling speed 300 rpm. The milling was carried out in 5 cycles of 10 min each with a 5 min pause. At each cycle, two drops of EtOH/H2O solution (1:1 *v*/*v*) were added. The samples were dried for 24 h. The triple complex was obtained through ball milling by adding citric acid to the already formed complex of β-CD and ibuprofen. The powders were mixed in a molar ratio of 1:1 (β-CD-IBU/CA). The milling conditions were 300 rpm. A total of 5 cycles of 10 min each with a 5 min pause were performed. The motivation for this order of interaction is the aim to increase the solubility of ibuprofen in an aqueous solution by initially dissolving the CA from the ternary complex and releasing the binary β-CD/IBU complex into solution and subsequently discharging the drug molecule from the cyclodextrin. Therefore, first, β-CD/IBU complex was formed by ball milling. For this purpose, the binding conditions established in our previous work were used [32], and a high degree of interaction between the two molecules was achieved. Figure 5 shows the DSC curves of pure IBU and of the β-CD/IBU complex. Judging from the enthalpy of IBU melting, ΔH^m^_IBU_, a reaction degree of nearly 90% completion was determined.

**Figure 6:**
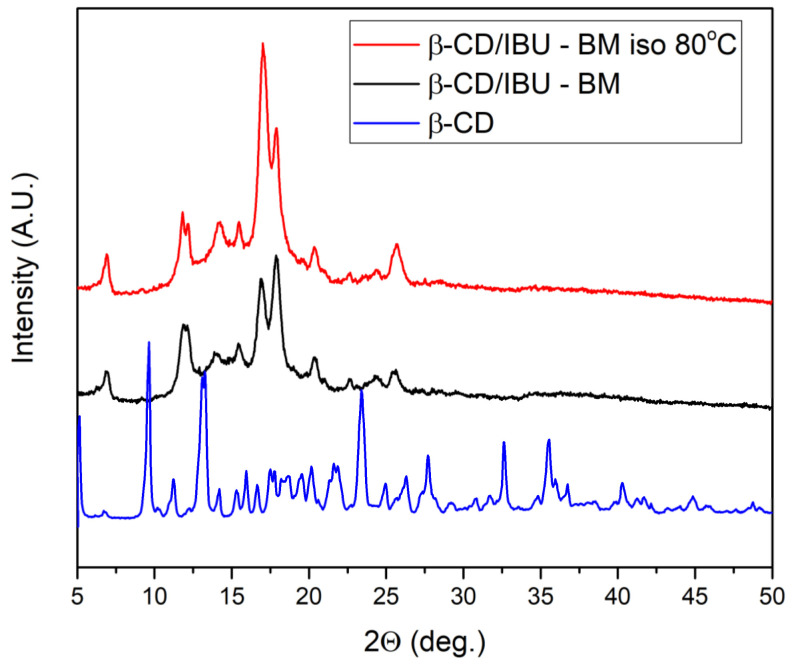
XRD patterns of β-CD/IBU after BM and BM followed by annealing.

An even more complete interaction (IBU inclusion into β-CD) was achieved after annealing the mechanically milled sample at 80 °C for 1 hour (Figure 5). This was evidenced by the thermogram which displayed the complete disappearance of the endothermic peak associated with IBU melting. XRD confirmed the formation of the complex as well as the small microstructural difference before and after thermal treatment of the new complex obtained by BM, as shown in Figure 6. Furthermore, our previous study proved that the IBU molecule is half located inside the cavity of β-CD [32].

**Figure 7:**
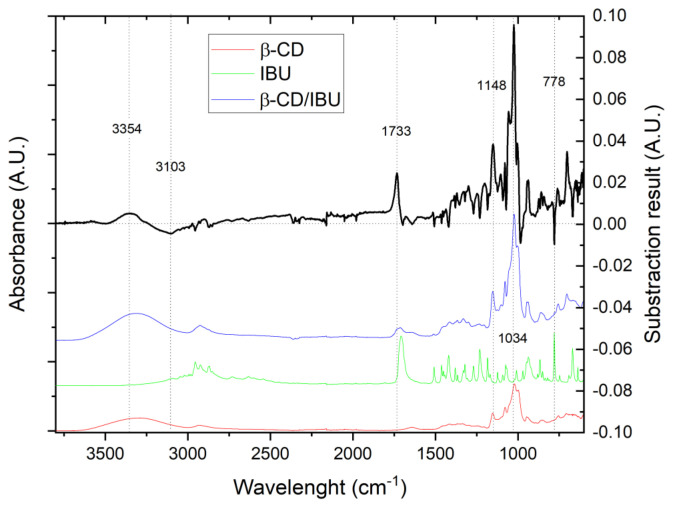
Spectra of inclusion complex of IBU in β-CD (top curve) and its ingredients (bottom curves).

This result was confirmed by FTIR in the present synthesis as well, as shown in Figure 7, which shows the spectra of the starting substances, the spectrum of the resulting complex, and the spectrum obtained by spectral subtraction [1]. Figure 7 shows clear bands at 3354, 3103, and 1733, as well as 1148 cm^−1^, which are characteristic of complex formation. The presence of the band at 1034 cm^−1^, with its high intensity and the change in spectral shape, clearly suggests that the guest molecule is located in the β-CD cavity, a result consistent with our previous observations [1]. The narrow characteristic band at ~778 cm^−1^ indicates that the benzene ring of the IBU molecule is also located inside the β-CD. A close examination of the spectral data presented in Figure 7 (black curve) reveals the characteristic spectral changes of the complex formation bands at ~1728 and at 3568 and 3345 cm^−1^. Here, the intensity of the spectral band at about 1027 cm^−1^ is reduced, as it is shifted. This is most likely due to a displacement of the IBU molecule from the interior of the β-CD and the complex formed, but the presence of the characteristic band at ~780 cm^−1^ and intensity of the band at 1027 cm^−1^ suggests that this process is only partial.

**Figure 8:**
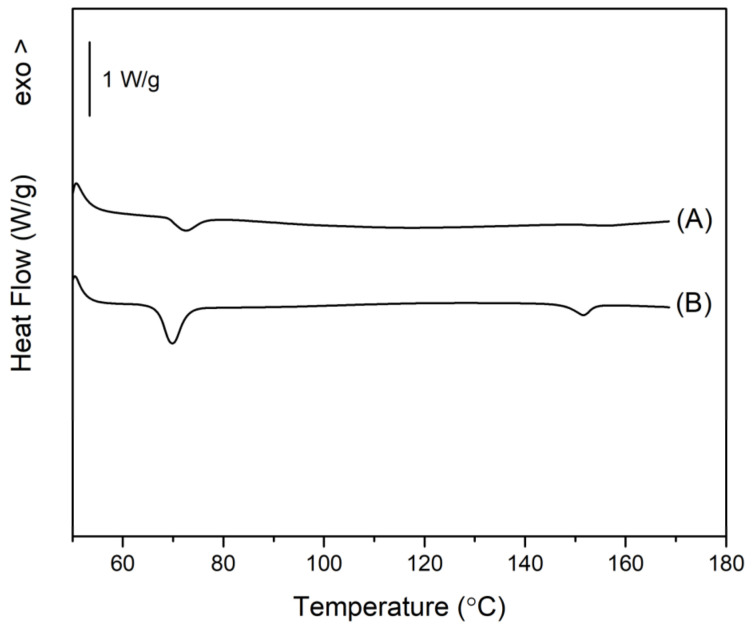
DSC scan of β-CD/IBU/CA formed by ball milling (A) and thermal annealing (B).

The next step, following the goal of the present study, was aimed at achieving an interaction between β-CD, IBU, and CA when all three compounds were ball milled together. DSC analysis of the resulting composites is presented in Figure 8, where the melting peaks of IBU and CA are strongly reduced or almost lacking (that of IBU is reduced to 15% of ΔH^m^ of pure IBU). Simultaneously, a mechanical mixture of the three components was annealed isothermally for 2 h at 80 °C under continuous stirring with a magnetic stirrer. The result was similar to that of BM, with the difference that some unbound CA still remained in the annealed sample, as shown in Figure 8.

**Figure 9:**
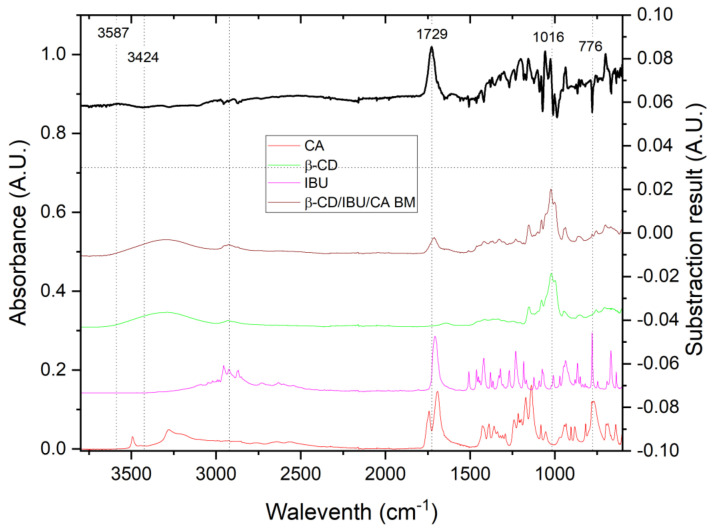
IR spectrum and differential spectrum of β-CD/IBU/CA formed by ball milling and spectra of the source substances from which the inclusion complexes were obtained.

The interaction between the three compounds was also confirmed by IR spectroscopy, as presented in Figure 9. The figure also includes the differential spectra of complexes formed by ball milling and thermal annealing and the substances from which they are prepared. Both spectra show the features of new complex formation due to the change in intensities and positions of the -OH and -COOH bands, around 3587 and 3424 and 1720–1730 cm^−1^, respectively. Because of the multicomponent system and the presence of more than one guest molecule, it is difficult to estimate the structure of the complexes from these data only. What is evident from the IR spectra of the simultaneously (three molecules) ball milled compositions is the presence of clear interaction between them, being however difficult to ascertain whether CA or IBU is located inside the β-CD cavity or bound from the outside. However, from the absence of the broad endothermic DSC peak, associated with the release of internally located H_2_O molecules, it can be argued that the β-CD cavity is occupied (partially or completely) by one of the two molecules (CA or IBU). Furthermore, we have learned from our previous studies that both IBU and CA enter the β-CD cavity (fully or partly) when each of them is milled separately with β-CD [1,32]. It is also worth recalling here that our theoretical study showed a little advantage of CA in competing with IBU for the hydrophobic cavity of β-CD, Figure 2.

**Figure 10:**
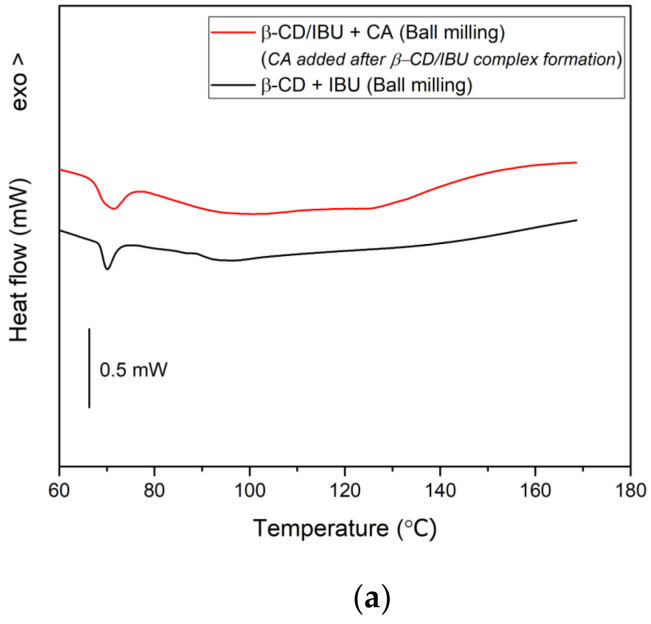

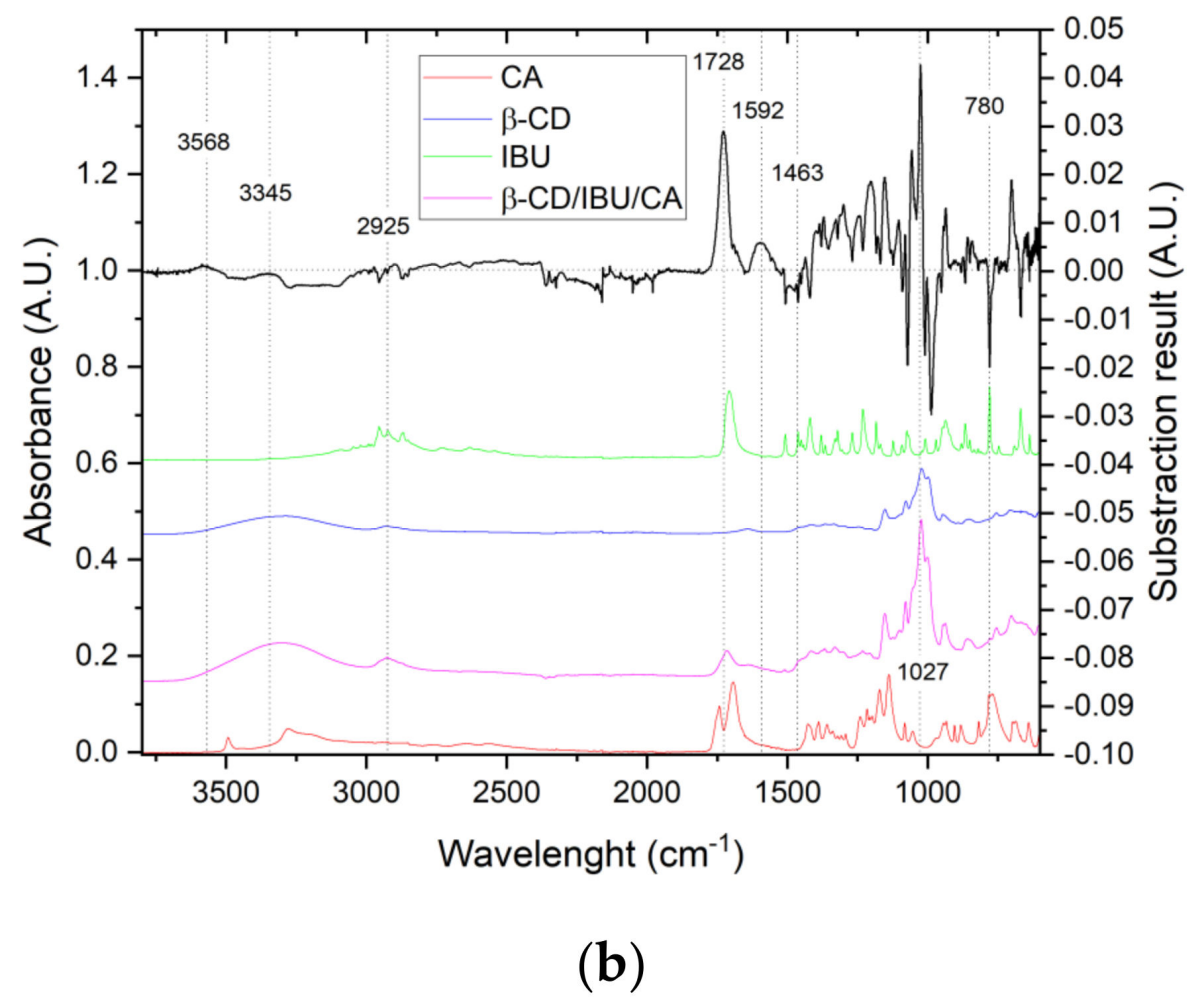
DSC scan of ball milled β-CD/IBU and β-CD/IBU/CA composites prepared in two successive steps (**a**) and IR spectrum of the complex formed by initial incorporation of IBU into β-CD, followed by successive binding with CA (**b**).

From the theoretical and experimental studies carried out so far and in line with the main objective of this work to provide enhanced solubility of IBU, a two-step synthesis consisting of first inclusion of IBU into β-CD and then binding the resulting β-CD/IBU complex with CA is imposed. The DSC curves of the as-formed ternary complexes are presented in Figure 10a and confirm the absence of “free” (unbound) CA as well as showing a strongly reduced melting peak of IBU, corresponding in melting enthalpy to the thermal curve of the binary β-CD/IBU complex. This result was confirmed by the IR spectroscopic study, which further showed that the most probable binding of CA to β-CD/IBU is via hydrogen bonds with the hydroxyl groups located outside the cyclodextrin cavity, Figure 10b.

To verify the solubility (equilibrium) and dissolution rate of CD and IBU after complexation with CA, a pharmacopeial (USP/Ph.Eur.) dissolution test was conducted. A threefold higher solubility of cyclodextrin was found when it was formed in a complex with citric acid (compared to that of pure β-CD). Specifically, it increased from 1.10^−4^ M to 3.10^−4^ M. A significant difference was also observed in the dissolution rate of IBU when incorporated into the β-CD/IBU/CA complex compared to that of pure ibuprofen. This result confirms the original idea of the study, namely, to significantly increase the solubility of ibuprofen by forming a two-phase system with CA.

**Figure 11:**
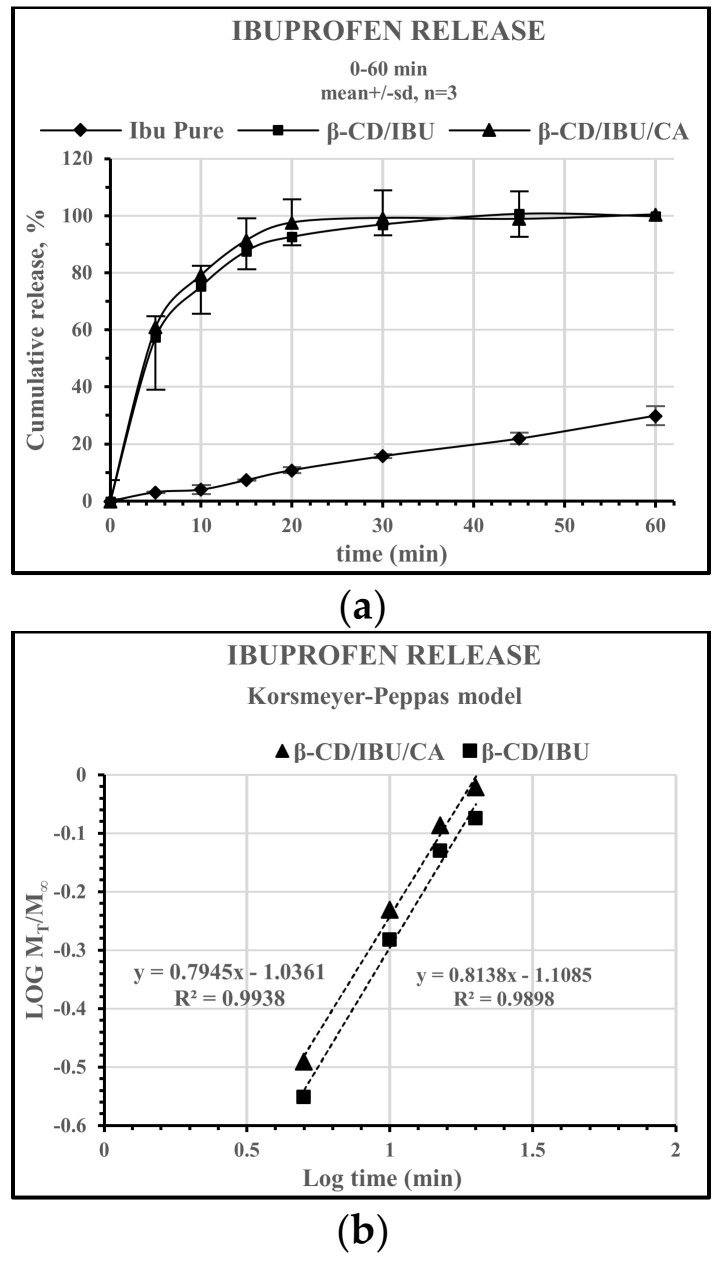
Dissolution rate of pure IBU, β-CD/IBU and β-CD/IBU/CA complexes (**a**) and fraction of IBU as a function of time in linear coordinates according to the Korsmeyer–Peppas model for the binary and ternary complexes (**b**).

After an initial burst up to the 5th min, ibuprofen is released in a fast manner from both β-CD/IBU and β-CD/IBU/CA until the 20th mn, with 93% and 98% dissolved, respectively (Figure 11).

The profiles of the two complexes are statistically equal with a similarity factor f2 = 70 and difference factor f1 = 3. Regardless of the kinetics, the ternary complex still exhibits a slightly higher release rate compared to the binary complex.

It can be seen from Table 1 that R^2^, which represents a qualitative index of linear correlation of the applied kinetic model, indicates that both β-CD/IBU and β-CD/IBU/CA release profiles best correspond to Korsmeyer–Peppas model (Equation (1)).

In order to calculate **n**, we used the part of the release curve corresponding to 60% of the loaded quantity of ibuprofen, i.e., M_t_/M_∞_ < 0.6. The model was plotted as log cumulative percentage drug release versus log time. As far as Fickian diffusion is indicative of molecule transport where boundaries are not presented, the only expected transport path is through the small and large β-CD openings/rims. Consequently, the number of hydrogen bonding between the β-CD, citric acid, ibuprofen, and water within β-CD cavity should be responsible for the rate of diffusion if we assume constant conditions in the gastrointestinal tract. Although the β-CD/IBU and β-CD/IBU/CA profiles differ slightly in their experimental values, **n**_β-CD/IBU_ = 0.3451 and **n**_β-CD/IBU/CA_ = 0.3535, we demonstrate a Fickian diffusion/release mechanism.

F_t_ = M_t_/M_∞_ = kt^n^, (1)

where F is fraction of drug released at time t; M_t_/M_∞_ is the fraction of ibuprofen released at the time t; k is kinetic release rate constant; n is diffusion or release exponent; t is time in minutes. The release exponent, n, and the release constant, k, are found by the linearization of Equation (1) in coordinates logM_t_/M_∞_ vs. logt (Figure 11b).

The release constant, k, mostly provides information about the formulation type and composition, such as the structural features of the cavity and surface of a β-CD structure, while the value of n is related to the drug release mechanism. When n is <0.45, the release pattern follows a Fickian diffusion mechanism. When 0.45 < n < 0.89, drug release follows anomalous transport behavior (non-Fickian transport). When n = 0.89, the diffusion process is characterized as Case II transport. However, when n > 0.89, it is termed as super Case II transport. Although the β-CD/IBU and β-CD/IBU/CA profiles differ slightly, their release kinetics follow an equal Fickian diffusion release mechanism.

## 3. Materials and Methods

β-cyclodextrin, ibuprofen, and citric acid used in the present study were of analytical grade, having a purity of 99.9%. β-cyclodextrin was purchased from Wacker Chemie AG, Germany, and ibuprofen from Teva Pharmaceuticals.

To prepare binary and ternary complexes, ibuprofen and β-cyclodextrin, as well as ibuprofen, β-cyclodextrin, and citric acid, were mixed in a molar ratio 1:1 (resp. 1:1:1) in dry conditions at room temperature. The mixtures were further used for all the mechanical milling experiments aiming to form the complexes. Some of the complexes formed by milling were further annealed in the DSC at 80 °C for 1 h to achieve more complete complexation.

The thermal behavior of complexes as well as of the pure substances was characterized by differential scanning calorimetry (DSC250-TA) under pure N_2_ atmosphere. Scanning rate of 10 °C/min was applied for the heating/cooling runs. The structure of the cyclodextrin, ibuprofen, and citric acid, as well as their complexes, was studied by X-ray diffraction using Cu-Kα radiation (Bruker D8).

ATR-FTIR spectra of β-CD, ibuprofen, and CA and the spectra the formed complexes between them were recorded on Agilent, Cary 670 FTIR spectrometer combined with Pike ATR accessory. The analyses were completed by direct deposition on a diamond ATR crystal at resolution of 2 cm^–1^ in the middle IR region (600–4000 cm^–1^). Due to the superposition of the component bands, the analysis of complex mixtures by IR spectroscopy can be challenging. Hence, a mathematical procedure for spectra subtraction was applied, described in detail in our recent study [1].

Dissolution testing was performed using USP/Ph.Eur. Type II [36,37] dissolution tester (paddle method) Erweka^®^ DT 820 at 37 °C and stirring speed 50 rpm. The dissolution medium used consisted of 500 mL of a solution with pH 1.2, prepared using 0.1 M HCl and supplemented with NaCl as per the guidelines outlined in the European Pharmacopoeia general text 5.17.1. The equivalent amount of 20 mg ibuprofen from each formulation (pure ibuprofen mixture with βCD and ternary complexes with βCD and CA) was tested for 60 min. Samples were taken at different time intervals as indicated, at 5th, 10th, 15th, 20th, 30th, 45th, and 60th minutes, and were analyzed after filtration (0.45 µm glass filers). UV-VIS spectroscopy analysis was performed with HALO DB-20S (UV-VIS Double beam spectrophotometer). The concentration of ibuprofen was determined at ƛ = 222 nm. The developed method was validated according to ICH guidelines with respect to specificity, linearity, limit of detection, limit of quantitation, accuracy, and reproducibility. Results graphically represent the amount of ibuprofen released as mass % [*w*/*w*] over the time of analysis (0–60 min.).

Statistical model independent (e.g., difference factor (f1), similarity factor (f2)) and model dependent methods were applied, aiming to evaluate the similarities between dissolution/release behaviors [38,39]. Criteria for acceptance are well described and harmonized by the international authorities [40,41]. F1 values up to 15 (0–15, dimensionless) and f2 values greater than 50 (50–100, dimensionless) demonstrate acceptable levels of similarity. In the present study, we apply zero order, first order, Higuchi, Hixson–Crowell, and Korsmeyer–Peppas as model-dependent methods [40,42].

## 4. Conclusions

Ternary β-CD/IBU/CA complexes have been prepared by ball milling following two pathways: (i) simultaneous milling of the three substances and (ii) applying a two-step procedure consisting of first drug inclusion into the cyclodextrin and then binding the formed β-CD/IBU complex with CA. Low-temperature heat treatment was also applied in some cases to ensure a more complete interaction between the components. The formation of the complexes was reliably confirmed by thermal analysis, X-ray diffraction, and IR spectroscopy. The experimental observations were also accompanied by a theoretical evaluation of the binding mechanisms between the three molecules as well as of the competition between CA and IBU for incorporation into the β-CD cavity. It was found that CA exhibits (slightly) higher affinity toward the host β-CD than its ibuprofen counterpart, evidenced by lower interaction ΔG for the former than the latter. Although CA is a bit more competitive than IBU for occupying the β-CD cavity, the calculations suggest that if the reaction takes place in a stepwise manner, then IBU is introduced first in the reaction medium, followed by the addition of CA, a binary β-CD/IBU construct, which will be formed first, followed by coordination of CA to the exterior of the complex.

The ternary complex thus formed, consisting of β-CD (with ibuprofen incorporated into its cavity) and citric acid, exhibited notably improved dissolution kinetics in water compared to the pure ibuprofen and almost the same compared to the binary β-CD/IBU system. It must, however, be stressed that a possible decrease in the needed β-CD quantity to solubilize hydrophobic drugs (such as ibuprofen) in pharmaceutical formulations can further attract safety and economic benefits for the industry.

## Figures and Tables

**Table 1:** Regression constants for different mathematical models for β-CD/IBU and β-CD/IBU/CA.

	Zero Order	First Order	Higuchi Model	Hixson-Crowell Model	Korsmeyer–Peppas
R^2^_β-CD/IBU_	0.9438	0.9157	0.9795	0.863	0.9898
R^2^ _β-CD/IBU/CA_	0.9540	0.9157	0.9859	0.7842	0.9938

## Data Availability

Data are contained within the article.
